# 

**Published:** 1902-10

**Authors:** 


### PERSONALS.

Dr. Roscoe R. Bell, of Brooklyn, N. Y., was selected to act as official veterinarian to the Bay Shore (L. I.) Horse Show, which took place in August last.

Dr. Geo. R. White, of Nashville, Tennessee, had the misfortune to have had his baby bitten by a rabid dog on June 3, the animal dying on the 6th. The doctor and Mrs. White took their child to the Pasteur Institute, St. Louis, for the eighteenth-day treatment, and there is every reason to believe that all danger has now passed.

Dr. R. O. Hasbrouck, of Passaic, N. J., spent ten days away from his practice in September, visiting parts of his own State and Eastern Pennsylvania.

Dr. W. G. Benner, of Doylestown, Pa., has passed the Civil Service examination for meat-inspector, and will shortly enter the service of the Bureau of Animal Industry.

Dr. D. F. Luckey, State Veterinarian of Missouri, spent a couple of weeks among the Farmers' Institutes in the early part of September.

Dr. C. T. Goentner, of Bryn Mawr, Pa., has become President of the bank at that place.

Dr. A. H. Baker, of Chicago, Illinois, was taken seriously ill at the A. V. M. A. Convention in Minneapolis, and was confined to his bed for a week subsequent to his return home.

Dr. E. G. Gilbert, of Pottstown, Pa., lost by theft in September veterinary instruments to the value of fifty dollars.

Dr. Chas. E. Cotton, of Minneapolis, Minnesota, spent several days in bed subsequent to the convention of the A. V. M. A. in his city. Charley, as his friends are wont to call him, proved a royal entertainer of his many friends at the convention.

State Veterinarian Pearson, of Pennsylvania, addressed the farmers' picnic of Chester County the latter part of September.

Dr. M. H. Reynolds, of St. Anthony Park, Minnesota, was seriously indisposed subsequent to the A. V. M. A. Convention. His assiduous attention to the welfare of the visitors to the A. V. M. A. Convention we fear proved too severe a strain.

Dr. W. L. Zuill, of Philadelphia, Pa., has gone to the sunny clime of Pasadena, California, where he contemplates locating permanently and to devote his entire attention to eye practice.

